# Changes in Migratory Speed Rate of Human Dental Pulp Stromal Cells Cultured in Advanced Platelet-Rich Fibrin

**DOI:** 10.1055/s-0042-1743146

**Published:** 2022-04-18

**Authors:** Anggraini Margono, Dini Asrianti Bagio, Indah Yulianto, Siti Utami Dewi

**Affiliations:** 1 Department of Conservative Dentistry, Faculty of Dentistry, Universitas Indonesia, Jakarta, Indonesia; 2 Department of Dermato Venereology, Faculty of Medicine, Universitas Sebelas Maret, Solo Surakarta, Indonesia; 3 Conservative Dentistry Residency Program, Faculty of Dentistry, Universitas Indonesia, Jakarta, Indonesia

**Keywords:** platelet-rich fibrin, dental pulp, stromal cell, cell migration assay

## Abstract

**Objective** Migratory speed rate evaluation of human dental pulp stromal cells (hDP-SCs) is one of the important steps in dental pulp regeneration. Therefore, the aim of the study is to analyze various concentrations of advanced platelet-rich fibrin (A-PRF) culture media toward hDP-SCs' migratory speed rate evaluations.

**Materials and Methods** The hDP-SCs were divided into four groups: control: hDP-SCs in Dulbecco's modified Eagle medium + 10% fetal bovine serum group; hDP-SCs in 1% A-PRF group; hDP-SCs in 5% A-PRF group; and hDP-SCs in 10% A-PRF group, which were planted in 24-well (5 × 10 ^4^ cell/well). The migratory speed rate of all groups was measured by using cell migration assay (scratch wound assay) after 24 hours. Cell characteristics were evaluated under microscope ( *Inverted microscope* , *Zeiss* , *Observer* Z1, UK) that can be read through *image-J* interpretation. This image J represented the measurement of migratory speed rate (nm/h) data. Statistical analysis was conducted using one-way analysis of variance and post hoc Tamhane's test ( *p* < 0.05) (IBM SPSS Statistics Software, version 22.0).

**Results** There was a statistically significant difference in the migratory speed rates of hDP-SCs among various concentration groups of A-PRF (1, 5, and 10%) compared with the control group.

**Conclusion** The increase in the migratory speed rate of hDP-SCs was highest in 10% A-PRF group.

## Introduction

Migration is a fundamental cellular behavior that plays an important role in homeostatic tissue maintenance and regeneration of injured organs and tissue, particularly contributing to several physiological processes, including vascular development and angiogenesis, tumorigenesis, wound healing, tissue engineering, and biomaterial design. 1 2 Cell migration plays an important role when cells must reach the injury site in a given environment to function, which can be assessed by several methods, such as scratch assay, transwell assay, and fluorescence microscopic assay. 3 4 The result of the assay is quantified using a single metric or a combination of metrics to have specific outcomes. This process begins with the host endogenous chemotaxis toward the injury site through biological signaling molecules. 5 Several types of signaling molecules have been evaluated *in vitro* , such as the stromal cell-derived factor-1a, basic fibroblast growth factor, vascular endothelial growth factor (VEGF), and platelet-derived growth factor (PDGF), which promote angiogenesis in the root canal, induce migration of stem cells, and promote mineralization. 5 6

Recruitment of dental pulp stem cells (DPSCs) is a prerequisite for regeneration of dentin damaged by severe caries and/or mechanical injury. Understanding the complex process of DPSCs' recruitment will benefit future *in situ* tissue engineering applications based on the stimulation of endogenous DPSC for dentin pulp regeneration. The current known mobilization signals and subsequent migration of DPSC toward the lesion site, which is influenced by the pulp inflammatory state and the application of pulp capping materials, are reviewed. 7 8 The migration of DPSC to the injured pulp site is a complex procedure that involves many processes that are not fully explained yet. These signals are influenced by the local microenvironment defined by caries, the inflammatory state, mechanical injury, and the application of restorative materials. 7 9

A recent trend in the domain of regenerative endodontics involves the use of autologous platelet concentrates as a source of growth factor (GF), which has shown promising clinical and radiographic results. 10 11 12 Second-generation platelet concentrate named by platelet-rich fibrin (PRF) was developed in 2001, and it has shown considerable advantages over platelet-rich plasma (PRP) in its application in regenerative endodontics. 13 PRF serves as a reservoir of bioactive molecules to support wound healing and bone regeneration. Although the cellular mechanisms by which PRF supports the clinical outcomes remain unclear, *in vitro* research provides possible explanations. Many previous studies suggested that PRF induces cell proliferation, migration, adhesion, and differentiation along with possessing its anti-inflammatory properties, further supporting its therapeutic potential in wound healing and bone regeneration. 14 15 According to several studies, PRF can act as a regulator of the immune system, and stimulate the wound healing process through various GFs such as PDGF-bb, VEGF, transforming growth factor (TGF)-b1, interleukin (IL)-1β, IL-6, IL-4, and tumor necrotic factor-a. 16 17 18

Ghanaati et al also produced an improved PRF form, which contains greater numbers of white blood cells, and named it advanced PRF (A-PRF). 16 19 Leukocytes have shown to be very important immunocytes capable of directing various cell types in the wound healing process. It has recently been assumed that an increase in leukocyte counts in the PRF matrix can be achieved by modification of methods, times, and speed of centrifugation, particularly from 2,700 rpm for 12 minutes to 1,500 rpm for 14 minutes, which can create a new type of PRF named by A-PRF. 19 It has been said that in accordance to this hypothesis, the release of several GFs in A-PRF was found significantly higher compared with that in lysate PRF (L-PRF) and PRF. 17 18 19 It has also been demonstrated by Huang et al that PRF can increase the proliferation and differentiation of dental pulp cells (DPCs) by upregulating of osteoprotegerin and alkaline phosphatase. These findings might serve as a basis for preclinical studies that address the role of PRF in reparative dentin formation. 20

Recently, modification of methods, times, and speed of centrifugation has resulted into several types of PRF, one example of which is L-PRF, which is made through a combination of freezing and thawing process. 16 17 18 21 Saeed et al studied the efficacy of human PRF (hPRF) exudate (compression without freeze and thaw methods) and showed that 10% of hPRF is the optimum concentration for human DPSCs' (hDPSCs) proliferation, but did not show the efficacy for migration of hDPSCs as the process that led to proliferation. 22 Same result was showed in previous studies by Illmilda et al 23 and Bagio et al. 24 Therefore the aim of this study was to analyze the effect of various concentrations of A-PRF conditioned media (CM) on migratory speed rate of human dental pulp stromal cells (hDP-SCs).

## Materials and Methods

### Human Dental Pulp Stromal Cell Culture

This study's protocol was approved by Ethical Committee of Faculty of Dentistry Universitas Indonesia (no.82/ *ethical approval* /FKGUI/IX/2019, protocol number: 070940819). This study is an *in vitro* study conducted at Prodia Stem Cell Laboratory by using hDP-SCs at fifth and sixth passages. Serum starvation procedure was conducted for 24 hours in Dulbecco's modified Eagle medium (DMEM; Thermo Fisher Scientific Inc., Massachusetts, United States) supplemented with 1% fetal bovine serum (FBS; Gibco, New York, New York, United States) reduced into 1% concentration.

### Preparation of Advanced Platelet-Rich Fibrin (A-PRF)

A-PRFs were taken from six healthy donors; 10-mL blood from vena cubitus was collected without anticoagulants in 5-mL vacuum blood collection tubes by a certified laboratory assistant (PT. Prodia Stem Cells (Prostem) Laboratory, Salemba Raya, Central Jakarta). In less than 2 minutes after collection, the blood samples were centrifuged at 1,500 rpm for 14 minutes to obtain an A-PRF gel layer separated from red blood cells. Then, A-PRF CM was incubated at 4°C for 24 hours and diluted to 1, 5, and 10% A-PRFs with DMEM. DMEM (Thermo Fisher Scientific Inc., Massachusetts, United States) supplemented with 10% FBS ( *Hyclone* , Global Life Science, United States) was used as positive control.

### Effect of Condition Medium on hDP-SCs' Migration Using Scratch Wound Assay

After serum starvation, hDP-SCs were divided into four groups: Control: hDP-SCs in DMEM + 10% FBS group; hDP-SCs in 1% A-PRF group; hDP-SCs in 5% A-PRF group; and hDP-SCs in 10% A-PRF group, which were planted in 24-well (5 × 10 ^4^ cell/well). The hDP-SCs were seeded in 24-wells plates, triplicate (triplo). Cells were mechanically disrupted with a sterile 200 μL pipette tip.

The migratory speed rate of all groups was measured by using cell migration assay (scratch wound assay) after 24 hours. Cell characteristic was evaluated under microscope ( *Inverted microscope* , *Zeiss* , *Observer* Z1, UK) that converted into *image-J* ( *image-J* , *Version 1.53k* , NIH) interpretation. Wound width was calculated as the average distance between the edges of the scratch by two observers and inter-rater reliability count with technical error of measurement. Speed rate was quantified by dividing the change in wound width by the time spent in migration.

### Data Analysis

Normality data analysis according to Shapiro–Wilk test ( *p* > 0.05) showed normal distribution data. Statistical analysis was performed by using one-way analysis of variance (ANOVA) and post hoc Tamhane test ( *p* < 0.05) (IBM SPSS Statistics Software, version 22.0).

## Result

**Fig. 1:**
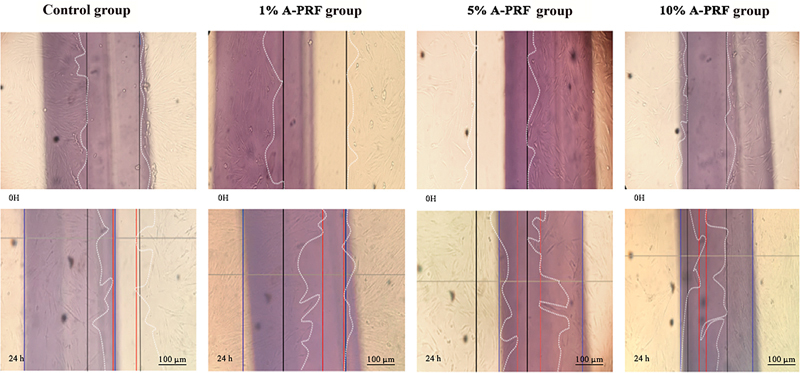
Migratory speed rate of human dental pulp stromal cells (hDP-SCs) between various advanced platelet-rich fibrin (A-PRF) groups (1, 5, and 10%) compared with control. There was a statistically significant difference between control and 5 and 10% of A-PRF groups ( *p* < 0.05), but there was no statistically significant difference between the control and 1% A-PRF group (* *p* > 0.05; n.s, not significant; post hoc Tamhane's test).

**Fig. 2:**
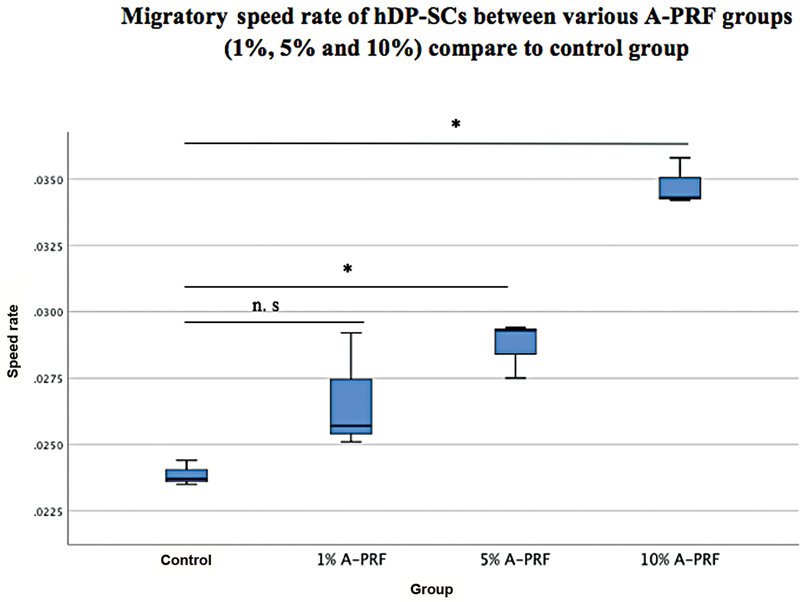
Qualitative data of human dental pulp stromal cells' migratory speed rate control and various advanced platelet-rich fibrin (A-PRF) groups (1, 5, and 10%) in 0 and 24 hours of observation (final wound showed with red line; cell movement demarcation showed with white dashed line).

There was statistically significant difference of migratory speed rate between all groups ( *p* < 0.05; one-way ANOVA test; Fig. 1 ). Post hoc Tamhane's analysis of migratory speed rate of A-PRF groups compared with control group is presented in Fig. 1 . The qualitative data of all groups are presented in Fig. 2 .

Post hoc Tamhane's analysis of migratory speed at various concentrations of A-PRF groups (1, 5, and 10%) showed that there was statistically significant difference between 10% A-PRF group and 1 and 5% A-PRF groups ( *p* < 0.05), but there was no statistically significant difference between 5% A-PRF and 1% A-PRF groups ( *p* > 0.05; Table 1 ).

**Table 1:** Post hoc analysis of migration speed rate of hDP-SCs cultured with 1, 5, and 10% A-PRF

Conditioned media	*p* -Value
1% A-PRF versus 5% A-PRF	0.815
1% A-PRF versus 10% A-PRF	0.000 a
5% A-PRF versus 10% A-PRF	0.000 a

Abbreviations: A-PRF, advanced platelet-rich fibrin; hDP-SCs, human dental pulp stromal cells.

a *p* < 0.05; post hoc Tamhane's test.

## Discussion

The hDP-SCs' recruitment depends not only on the soluble chemotactic signals, but also on their interaction with neighboring cells and the extracellular matrix. 25 26 Cells are able to sense and mount a response to physical and mechanical properties and forces. Soluble factors such as cytokines or GFs are sufficient to activate receptors and initiate migration signaling. Signal factor from the niche and microenvironment controls stem cells' behavior such as regulation of proliferation, division, and migration. A good microenvironment such as condition medium allows stem cells to perform physiological activities. 6 7 9 FBS is an animal serum supplement that is commonly used in culture media but can induce a xenogeneic reaction, besides can be contaminated with the bovine virus, and takes a long time to expand. FBS is the gold standard cell culture medium and has numerous GFs and low gamma globulins compared with other animal-derived supplements. Recently, the use of a human-derived serum, such as PRF, has been developed to replace FBS as a cell culture supplement. 27 28 This explains why in this study only DMEM was used as a control.

The use of modified PRF, named by A-PRF, is developing in recent years; this is due to the easier preparation of the PRF compared with PRP and the great result of GF releases. The result of this study also correlated with the previous research conducted by Ghanaati et al; that is, the modification of speed and time of centrifugation from 2,700 rpm for 10 minutes to 1,500 rpm for 14 minutes can increase the release of granulocytes of A-PRF that can then induce GFs of A-PRF. 19 The biological properties of A-PRF, which has a great number of leukocytes, and platelets and GFs promote both proliferation and differentiation. 17 18 The TGF-β, PDGF, VEGF, eotaxin, and CCL-5 released by the leukocytes promote local vascularization and tissue repairing, mainly due to the control of the inflammatory process by anti-inflammatory cytokines IL-4, IL-6, and IL-10. 16 19 29

This result showed there was statistically significant difference of migratory speed rate between all groups of 1, 5, and 10% A-PRF ( *p* < 0.05) after 24 hours of migration, which is the first step of wound healing. The increase in the migratory speed rate of hDP-SCs was highest in 10% A-PRF group as shown in Fig. 1 . These various concentration results were in line with previous study by Illmilda et al, who concluded that the highest proliferation rate was at 25% A-PRF, and the lowest was with 10% A-PRF on the first day of evaluation. 23

The hDPSC proliferation means did not significantly differ with 10, 20, or 25% A- PRFs on days 3 or 5. 23 The result of this study was in conjunction with the results of Saeed et al and Swastiningtyas et al, which showed a greater effect on low proliferation of L-PRF. 22 30 31 Saeed et al studied the efficacy of hPRF exudate (compression without freeze and thaw methods) and showed that 10% of hPRF is the optimum concentration for hDP-SCs' proliferation, but did not show the efficacy for migration of hDP-SCs. 22

The study by Utami Dewi et al showed that various concentrations of L-PRF have different effects on migration rate of DPSCs. The best migration rate of DPSCs was showed in 5% L-PRF after 24 hours compared with all groups (1% L-PRF, 10% L-PRF, 20% L-PRF, and 25% L-PRF). 30 Low concentration of L-PRF showed greater result in DPSCs' migration. Bagio et al proved that the ideal concentration of A-PRF, which have great potential ability of human DPCs' odontogenic differentiation, is at 1 to 5%. 24 From this result, various concentrations of A-PRF as well as L-PRF, as allogenic platelet concentrations, which are platelet-rich blood derivatives, have been widely used in stem-cell-based therapy as CM showed a great potential of stem cells' migration.

Beyond the confusion between migration and proliferation processes, based on result of the study by Saeed et al, it was showed that 10% hPRF has the optimum concentration for hDPSCs's proliferation only. 22 This phenomenon can be explained through the understanding of the concept of cell migration assay that disambiguates cell migration from cell proliferation. In traditional migration assays (scratch assay), cells are allowed to migrate into an initially cell-free exclusion zone. At the end of the migration period, cells located in the exclusion zone are simply enumerated. This traditional approach cannot distinguish cells that have physically crawled into the exclusion zone from cells that were “born” there, analogous to estimating national immigration rate by only measuring increases in population. 32

On the other hand, post hoc Tamhane's analysis of migratory speed rate between various concentrations of A-PRF groups (1, 5, and 10%) showed that there was statistically significant difference between 10% A-PRF and 1 and 5% A-PRF groups ( *p* < 0.05), but there was no statistically significant difference between 5% A-PRF and 1% A-PRF groups ( *p* > 0.05; Table 1 ). It can be concluded from this study that 10% A-PRF has the highest migratory speed rate among all concentration groups (1, 5, and 10% FBS as a control group). Based on microscopic evaluation, hDP-SCs migrate to the scratch area after 2 hours. This evaluation showed that 10% A-PRF has the shortest wound width compared with all groups after 24 hours of observation ( Fig. 2 ). These data were also supported by data showing that the highest migration speed was in the 10% A-PRF group, as shown in Fig. 1 . The wound area of all groups showed cells filled the wounded area with the best result at 10% A-PRF, whereas in other concentrations the groups showed the same result but not as good as 10% A-PRF ( Fig. 2 ) after 24-hour observation. This result can be explained based on a study by Akpinar et al, according to which hDPSCs in the G1 phase of the cell cycle showed the most significant number of cells (78%) compared with those in the S and G2 phases. 33 34 Our study was conducted to determine the ability of A-PRF to support hDPSC migration *in vitro* after 24 hours after plating with three concentrations.

In line with the migratory speed rate, various DPSC mobilization signals have been reported, which include several GFs, various chemokines, and the signaling pathways, which are promising targets for promoting dental stem cells' migration via modulating adhesion, autophagy, cytoskeleton rearrangement, and upregulating the expression of chemokines and their receptors, which need to be further investigated in the next continuous research. 34 35 Previous study proved that A-PRF releases a significant amount of GFs such as TGF-B, VEGF, and PDGF compared with the first generation of PRF that plays an important role in tissue regeneration and vessel formation, including dental pulp tissue. 36

The limitation of this study relies on the scratch wound assay method simply enumerated cells in the exclusion zone and therefore cannot distinguish the effects of proliferation towards migration. Therefore, further research is needed with new approaches by labeling cells with a fluorescent lineage tracing dye at the beginning of the assay; in this way migration can be evaluated in terms of the original or parental cell population.

## Conclusion

Various concentrations of A-PRF have different effects on migration rate of DPSCs. Increase in migratory speed rate of hDP-SCs was the highest in 10% A-PRF group, as well as showed in microscopic evaluation whereas after 24 hour observation has the shortest wound width compared with all groups. For the clinical application purposes, A-PRF can be used as suitable CM that can enhance niche biology for dental pulp homing cells in regeneration of the immature tooth.

## References

[1] Anh T MN Ha L BT Thuy A VP *In vitro* evaluation of proliferation and migration behaviour of human bone marrow-derived mesenchymal stem cells in presence of platelet-rich plasma Int J Dent 2019 9.63982E6 10.1155/2019/9639820 PMC6481138 31093287

[2] De Becker A Riet I V Homing and migration of mesenchymal stromal cells: how to improve the efficacy of cell therapy? World J Stem Cells 2016 8 03 73 87 27022438 10.4252/wjsc.v8.i3.73 PMC4807311

[3] Grada A Otero-Vinas M Prieto-Castrillo F Obagi Z Falanga V Research techniques made simple: analysis of collective cell migration using the wound healing assay J Invest Dermatol 2017 137 02 e11 e16 28110712 10.1016/j.jid.2016.11.020

[4] Pijuan J Barceló C Moreno D F In vitro cell migration, invasion, and adhesion assays: from cell imaging to data analysis Front Cell Dev Biol 2019 7 107 31259172 10.3389/fcell.2019.00107 PMC6587234

[5] Eramo S Natali A Pinna R Milia E Dental pulp regeneration via cell homing Int Endod J 2018 51 04 405 419 29047120 10.1111/iej.12868

[6] Iwasa S N Babona-Pilipos R Morshead C M Environmental factors that influence stem cell migration: an “electric field” Stem Cells Int 2017 2017 4.276927E6 10.1155/2017/4276927 PMC5447312 28588621

[7] Rombouts C Jeanneau C Bakopoulou A About I Dental pulp stem cell recruitment signals within injured dental pulp tissue Dent J (Basel) 2016 4 02 8 29563450 10.3390/dj4020008 PMC5851269

[8] Andreas K Sittinger M Ringe J Toward in situ tissue engineering: chemokine-guided stem cell recruitment Trends Biotechnol 2014 32 09 483 492 25059433 10.1016/j.tibtech.2014.06.008

[9] Chmilewsky F Jeanneau C Dejou J About I Sources of dentin-pulp regeneration signals and their modulation by the local microenvironment J Endod 2014 40(4, Suppl):S19 S25 24698688 10.1016/j.joen.2014.01.012

[10] Ray H L Jr Marcelino J Braga R Horwat R Lisien M Khaliq S Long-term follow up of revascularization using platelet-rich fibrin Dent Traumatol 2016 32 01 80 84 26095129 10.1111/edt.12189

[11] Bezgin T Yilmaz A D Celik B N Kolsuz M E Sonmez H Efficacy of platelet-rich plasma as a scaffold in regenerative endodontic treatment J Endod 2015 41 01 36 44 25459571 10.1016/j.joen.2014.10.004

[12] Narang I Mittal N Mishra N A comparative evaluation of the blood clot, platelet-rich plasma, and platelet-rich fibrin in regeneration of necrotic immature permanent teeth: a clinical study Contemp Clin Dent 2015 6 01 63 68 25684914 10.4103/0976-237X.149294 PMC4319348

[13] Naik B Karunakar P Jayadev M Marshal V R Role of platelet rich fibrin in wound healing: a critical review J Conserv Dent 2013 16 04 284 293 23956527 10.4103/0972-0707.114344 PMC3740636

[14] Strauss F J Nasirzade J Kargarpoor Z Stähli A Gruber R Effect of platelet-rich fibrin on cell proliferation, migration, differentiation, inflammation, and osteoclastogenesis: a systematic review of in vitro studies Clin Oral Investig 2020 24 02 569 584 10.1007/s00784-019-03156-9 PMC6988133 31879804

[15] Maria P P Amin S Mayya A Naik R Platelet rich fibrin in regenerative endodontics: an update Int J Appl Dent Sci. 2020 6 02 25 29

[16] Caymaz M G Uyanik L O Comparison of the effect of advanced platelet-rich fibrin and leukocyte- and platelet-rich fibrin on outcomes after removal of impacted mandibular third molar: a randomized split-mouth study Niger J Clin Pract 2019 22 04 546 552 30975961 10.4103/njcp.njcp_473_18

[17] Masuki H Okudera T Watanebe T Growth factor and pro-inflammatory cytokine contents in platelet-rich plasma (PRP), plasma rich in growth factors (PRGF), advanced platelet-rich fibrin (A-PRF), and concentrated growth factors (CGF) Int J Implant Dent 2016 2 01 19 27747711 10.1186/s40729-016-0052-4 PMC5005757

[18] Chatterjee A Debnath K Comparative evaluation of growth factors from platelet concentrates: an *in vitro* study J Indian Soc Periodontol 2019 23 04 322 328 31367128 10.4103/jisp.jisp_678_18 PMC6628779

[19] Ghanaati S Booms P Orlowska A Advanced platelet-rich fibrin: a new concept for cell-based tissue engineering by means of inflammatory cells J Oral Implantol 2014 40 06 679 689 24945603 10.1563/aaid-joi-D-14-00138

[20] Huang F M Yang S F Zhao J H Chang Y C Platelet-rich fibrin increases proliferation and differentiation of human dental pulp cells J Endod 2010 36 10 1628 1632 20850666 10.1016/j.joen.2010.07.004

[21] Risya D M Asrianti D Margono A The efficacy of platelet-rich fibrin lysate (PRF-L) for fibroblast cell proliferation J Int Dent Med Res 2017 10 809 813

[22] Saeed M A El-Rahman M A Helal M E Zaher A R Grawish M E Efficacy of human platelet rich fibrin exudate vs fetal bovine serum on proliferation and differentiation of dental pulp stem cells Int J Stem Cells 2017 10 01 38 47 28215057 10.15283/ijsc16067 PMC5488775

[23] Illmilda A D Margono A Julianto I Wardoyo M P Advanced platelet rich fibrin (A-PRF) supplemented culture medium for human dental pulp stem cell proliferation J Int Dent Med Res. 2019 12 02 396 400

[24] Bagio D A Julianto I Suprastiwi E Margono A Ideal concentration of advanced-platelet rich fibrin (A-PRF) conditioned media for human dental pulp stem cells differentiation Pesqui Bras Odontopediatria Clin Integr 2019 19 01 1 9

[25] Fu X Liu G Halim A Ju Y Luo Q Song A G Mesenchymal stem cell migration and tissue repair Cells 2019 8 08 784 31357692 10.3390/cells8080784 PMC6721499

[26] de Lucas B Pérez L M Gálvez B G Importance and regulation of adult stem cell migration J Cell Mol Med 2018 22 02 746 754 29214727 10.1111/jcmm.13422 PMC5783855

[27] Wen J Li H T Li S H Li X Duan J M Investigation of modified platelet-rich plasma (mPRP) in promoting the proliferation and differentiation of dental pulp stem cells from deciduous teeth Braz J Med Biol Res 2016 49 10 e5373 27599200 10.1590/1414-431X20165373 PMC5018690

[28] Piletz J E Drivon J Eisenga J Human cells grown with or without substitutes for fetal bovine serum Cell Med 2018 10 10 2.15517901875514E15 10.1177/2155179018755140 PMC6172986 32634183

[29] Miron R J Zucchelli G Pikos M A Use of platelet-rich fibrin in regenerative dentistry: a systematic review Clin Oral Investig 2017 21 06 1913 1927 10.1007/s00784-017-2133-z 28551729

[30] Utami Dewi S Margono A Asrianti D Fatmasari A Effects of various concentration of lysate platelet-rich fibrin on human dental pulp stromal cell migration activity Clin Oral Investig 2020 24 02 569 584

[31] Swastiningtyas S Margono A Asrianti D Oktayani R Yulianto I Analysis of lysate platelet-rich fibrin effects on human dental pulp stem cell differentiation through dentin sialophosphoprotein expression Int J App Pharm. 2020 12 02 34 37

[32] Glenn H L Messner J Meldrum D R A simple non-perturbing cell migration assay insensitive to proliferation effects Sci Rep 2016 6 31694 27535324 10.1038/srep31694 PMC4989229

[33] Akpinar G Kasap M Aksoy A Duruksu G Gacar G Karaoz E Phenotypic and proteomic characteristics of human dental pulp derived mesenchymal stem cells from a natal, an exfoliated deciduous, and an impacted third molar tooth Stem Cells Int 2014 2014 457059 25379041 10.1155/2014/457059 PMC4212660

[34] Yang J W Zhang Y F Wan C Y Autophagy in SDF-1α-mediated DPSC migration and pulp regeneration Biomaterials 2015 44 11 23 25617122 10.1016/j.biomaterials.2014.12.006

[35] Pan S Dangaria S Gopinathan G SCF promotes dental pulp progenitor migration, neovascularization, and collagen remodeling - potential applications as a homing factor in dental pulp regeneration Stem Cell Rev Rep 2013 9 05 655 667 23703692 10.1007/s12015-013-9442-7 PMC5023022

[36] Caruana A Savina D Macedo J P Soares S C From platelet-rich plasma to advanced platelet-rich fibrin: biological achievements and clinical advances in modern surgery Eur J Dent 2019 13 02 280 286 31509878 10.1055/s-0039-1696585 PMC6777161

